# Comparison of corifollitropin alfa and daily recombinant follicle-stimulating hormone in poor responder patients undergoing in vitro fertilization cycles

**DOI:** 10.4274/tjod.08634

**Published:** 2017-12-30

**Authors:** Süleyman Akarsu, Sibel Demir, Funda Gode, Ahmet Zeki Işık

**Affiliations:** 1 İzmir Medical Park Hospital, Clinic of Obstetrics and Gynecology, In Vitro Fertilization Unit, İzmir, Turkey; 2 İzmir Başkent University Hospital, Clinic of Obstetrics and Gynecology, İzmir, Turkey

**Keywords:** Corifollitropin alfa, diminished ovarian reserve, gonadotropin-releasing hormone antagonist, in vitro fertilization, poor responder

## Abstract

**Objective:**

The aim of this study was to compare the effect of corifollitropin alfa (CFA) and recombinant follicle-stimulating hormone (rFSH) in poor-responder patients undergoing antagonist cycles.

**Materials and Methods:**

The study was a retrospective analysis of the treatment results of 214 poor responder patients who had been admitted to the In Vitro Fertilization Unit of İzmir Medical Park Hospital between November 2014 and November 2016. Intracytoplasmic sperm injections were performed in 38 patients (group 1) with CFA, and the remaining 176 (group 2) with rFSH for controlled ovarian hyperstimulation.

**Results:**

The age, body mass index, anti-müllerian hormone level, duration of infertility, duration of induction and antral follicle number were similar in the two groups. There was no difference in the total aspirated oocyte counts, mature oocyte ratio, fertilization rate, implantation rate, and clinical pregnancy rates between the two groups. The implantation rate was 9/38 (23.6%) in group 1 and 42/176 (23.8%) in group 2, whereas the clinical pregnancy rates were 16.3% and 17.2%, respectively.

**Conclusion:**

No difference was found in terms of oocyte count, fertilization rate, implantation rate, and clinical pregnancy rates of CFA or rFSH use in the antagonist cycles in poor-responder patients.

## PRECIS:

No difference was found in terms of oocyte count, fertilization rate, implantation rate, and clinical pregnancy rates of CFA or rFSH use in the antagonist cycles in poor-responder patients.

## INTRODUCTION

Corifollitropin alfa (CFA) is a new gonadotropin analogue with follicle-stimulating hormone (FSH) activity, which is effective for 7 days at the beginning and continuation of multi-follicular development^(1)^. This FSH analogue is a recombinant molecule that contains the carboxy terminal peptide structure of the human FSH beta subunit, but does not exhibit luteinizing hormone (LH) activity that affects only FSH receptors^(1,2)^. The greatest advantage of this molecule is the half-life of about 68 hours^(3)^. The pharmacodynamic properties of this molecule are that the serum concentration reaches the peak level (T-_max_) in a short time and reaches the _max_imum concentration (C-_max_) after 25-45 hours of injection^(4)^. There are no adverse effects or complications associated with this drug, which is well tolerated by patients^(5)^. The treatment of patients with low over-exposure has been the main topic of many randomized studies in past years. Different treatment regimens have been applied to increase over-response and pregnancy rates. Decreasing oocyte quality and decreasing over-reserve are closely related to female age^(6,7,8)^. The European Society of Human Reproduction and Embryology developed a new definition called Bologna criteria in 2011 for the poor-response patient group^(9)^. These criteria are: 1) older age of women (40 years) or other existing factors causing diminished ovarian reserve (DOR); 2) less oocyte counts in treatments taken in previous treatments (3 oocytes); 3) disorder in over reserve tests [antral follicles <5-7 or anti-müllerian hormone (AMH) <0.5-1.1 ng/mL]. Two of these criteria will cause the patient to have a diagnosis of DOR^(9)^.

## MATERIALS AND METHODS

This study was the result of a retrospective study of 214 patients who were diagnosed as having DOR according to the Bologna criteria at the in vitro fertilization (IVF) Unit of İzmir Medical Park Hospital between November 2014 and November 2016. Ethics committee approval was obtained from İzmir University Ethics Committee (approval number: 012742) before the study commencement. Informed consent was obtained from all of the study participants. The inclusion criteria were: being aged <45 years old, regular menstrual cycles (24-35 days), body mass index of 18-30 kg/m^2^, absence of any endocrine pathology, no severe male factor (total progressive motile sperm count 1 million/mL (Table 1). Thirty-eight patients were treated with single-dose subcutaneous injection of 150 µg CFA (Elonva, NV Organon, Oss, Netherlands) on the second or third day of the cycle as the first day of controlled ovarian hyperstimulation (COH). On the seventh day of treatment, 300 IU of highly purified human menopausal gonadotrophin (Merional, IBSA, Switzerland or Menopur, Ferring, Turkey) were administered, similar to the protocol of Polyzos and Devroey^(6)^, subcutaneously per day to each patient (group 1). The remaining 176 patients were treated with 300 IU follitropin alpha (Gonal-f; Merck, Switzerland) or follitropin beta (Puregon; NV Organon, Oss, Netherlands) subcutaneously on the second or third day of the cycle (group 2). In both groups, 0.25 mg gonadotropin-releasing hormone (GnRH) ganirelix (Orgalutran; NV Organon, Oss, Netherlands) or cetrorelix (Cetrotide; Merck, Switzerland) was given daily until the day of human chorionic gonadotropin (hCG) to prevent premature luteinization when the follicle diameter was 13 mm or more. When the leading follicle was 18 mm, 250 µg recombinant hCG (Ovitrelle; Merck, Switzerland) was administered subcutaneously for the final maturation of the oocyte. Thirty-five hours later, under general anesthesia, transvaginal ultrasound-guided oocyte pick-up was performed. Intracytoplasmic sperm injections was applied to all patients. All embryos were cultured for three days in vitro and then transferred in the presence of transabdominal ultrasonographic guidance. Four hundred milligrams per day of micronized progesterone (Progestin capsules 200 mg, Koçak Farma, İstanbul, Turkey) and 90 mg progesterone gel (Crinone gel, Merck, Switzerland) were applied vaginally for luteal phase support. On the second day of the cycle, serum estradiol, LH, and FSH, and on the day of hCG treatment, estradiol, LH, and progesterone values ​​were recorded. On the second, seventh, and hCG days of the cycle, follicle evaluation was performed using transvaginal ultrasonography. Twelve days after the embryo transfer, a beta-hCG test was performed. Transvaginal ultrasonographic evaluation was performed for fetal heart beat two weeks after a finding a positive hCG test.

### Statistical Analysis

The Statistical Package for the Social Sciences program (SPSS 20) [International Business Machines (IBM) Corp. released 2011. IBM SPSS Statistics for Windows, version 20.0, Armonk, NY: IBM Corp.] was used to evaluate the data. Variables mean + standard deviation and median (minimum-maximum) percentage and frequency values ​​were used. The homogeneity of the variances from the preconditions of the parametric tests was checked using the Levene test. The assumption of normality was examined using the Shapiro-Wilk test. The differences between the two groups were evaluated using Student’s t-test when parametric test prerequisites were provided, and the Mann-Whitney U test was used when the conditions were not met. Categorical data were analyzed using the maximum likelihood and chi-square tests. In cases where the expected frequencies were less than 20%, the Monte Carlo Simulation Method was used including these frequencies in the analysis. For the significance level of the tests, p<0.01 was accepted.

## RESULTS

CFA (group 1) was applied to 38 of 214 patients with DOR, as diagnosed according to the Bologna criteria, and recombinant FSH and antagonist protocol (group 2) treatment was applied to the remaining 176 patients. It was not possible to retrieve any oocytes in 13 (34.2%) patients in group 1 and 57 (32%) patients in group 2; therefore, these cycle were cancelled (p=0.718). The mean number of oocytes collected in group 1 with 25 patients with at least one oocyte was 3.2, and in group 2 with 119 patients it was 3.4 (p=0.879). The mean number of mature oocytes metaphase 2 was 1.8 in group 1 and 1.6 in group 2 (p=0.745). The duration of stimulation was 10.52 days in group 1 and 10.34 days in group 2 (p=0.894). The mean number of transferred embryos was 1.6 in group 1, whereas this value was 1.5 in group 2 (p=0.478). The implantation rate was 9/38 (23.6%) in group 1 and 42/176 (23.8%) in group 2 (p=0.578), and the clinical pregnancy rate was 16.3% and 17.2%, respectively (p=0.622). Multiple pregnancy and drug adverse effects were not observed in any patients (Table 2).

## DISCUSSION

Since 2008, there have been different results in a limited number of studies on single-dose CFA administration. In 2008, Devroey et al.^(10)^ wrote the first study about CFA. The authors suggested that a single injection of corifollitropin alfa induced a dose-related increase in multifollicular development and in the number of retrieved oocytes^(11)^. Devroey et al.^(10)^ postulated that CFA was a novel and effective treatment option for potential normal-responder patients undergoing ovarian stimulation with GnRH antagonist co-treatment for IVF resulting in ongoing pregnancy rates equal to that achieved with daily rFSH. Mahmoud Youssef et al.^(12)^ published a meta-analysis in 2012. They included four randomized trials involving 2326 women. There was no evidence of a statistically significant difference in ongoing pregnancy rates for CFA versus rFSH^(12)^. Boostanfar et al.^(13)^ designed a large comparative randomized double-blind trial that confirmed the non-inferiority of pregnancy rates for CFA compared with recombinant FSH in a GnRH antagonist COH protocol in advance-age patients undergoing IVF. CFA was proven noninferior to daily rFSH with respect to cardiopulmonary resuscitations, number of oocytes retrieved, and live birth rates, and the drug was generally well tolerated^(13)^. Another study was designed by Polyzos et al.^(14)^ with poor ovarian responders. In this study, the Bologna criteria were used to enroll the patients. The protocol with CFA in this group of patients resulted in low poor responder (PR) similar to the conventional short agonist protocol^(14)^. Revelli et al.^(15)^ demonstrated that starting CFA on day 4 of the cycle resulted in comparable PR with significantly less injections and a similar risk of Ovarian Hyperstimulation syndrome (OHSS)^(15)^. On the other hand Oehninger reported that, in women aged 35 to 42 years, the prediction of ovarian response to CFA treatment was related with AMH, AFC, and age at the start of stimulation for in both high and PR patients^(16)^. In addition, basal FSH values for high ovarian response and menstrual cycle length for poor ovarian response were prognostic. In a meta-analysis consisting of 2138 women who were randomized to receive corifollitropin alfa and 1788 who were randomized to receive daily rFSH, Fensore et al.^(17)^ emphasized that the risk of cycle cancellation due to overstimulation was significantly higher in the CFA group. On the other hand, the incidence of OHSS was comparable between patients receiving long-lasting or daily rFSH. Accordingly, they suggested that CFA resulted in a higher number of metaphase 2 oocytes collected and a higher number of cycles cancelled due to overstimulation; therefore, CFA should be cautiously considered in women with the potential of being hyper-responders. In view of this meta-analysis, one can consider using CFA more effectively for patients with DOR, but to date, the results have not been consistent. In a retrospective study, Polyzos et al.^(14)^ used HP-hMG as an additional gonadotropin in poor-responder patients according to the Bologna criteria. They achieved a very reasonable ongoing pregnancy rate (28%) in patients aged below 40 years, whereas no pregnancies occurred in patients aged over 40 years.

### Study Limitations

The retrospective design is the major limitation of our study. Therefore, prospective randomized studies are also necessary about this subject.

## CONCLUSION

CFA is a long-acting novel gonadotropin for COH. In our study, we used a similar protocol to Polyzos et al.^(14)^ who found considerably high PR in patients with DOR, particularly in the younger age group. Unfortunately, we could not show any difference between the two different COH modalities. Although the CFA treatment was well accepted by the patients, the high cost of the medication was a limiting concern. As far as we know, this is the first study to report the use of CFA in patients with DOR from our country.

## Figures and Tables

**Table 1:**
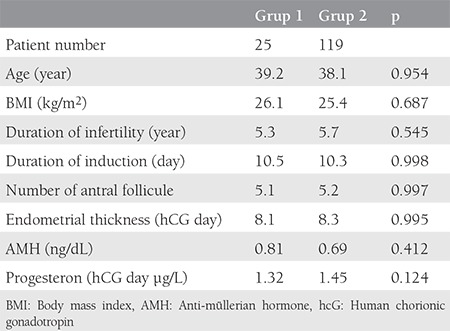
Demographic characteristics of patients

**Table 2:**
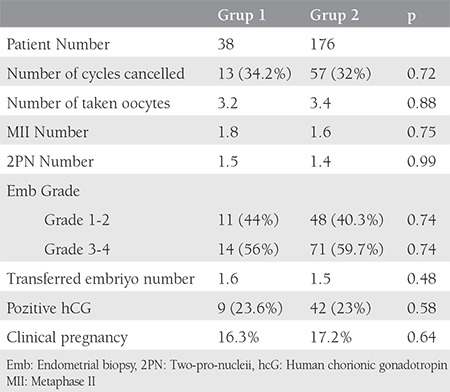
Over response, embryo results and pregnancy rates
